# DFMO/eflornithine inhibits migration and invasion downstream of MYCN and involves p27^Kip1^ activity in neuroblastoma

**DOI:** 10.3892/ijo.2013.1835

**Published:** 2013-02-21

**Authors:** DANA-LYNN T. KOOMOA, DIRK GEERTS, INGO LANGE, JAN KOSTER, ANTHONY E. PEGG, DAVID J. FEITH, ANDRÉ S. BACHMANN

**Affiliations:** 1 Department of Pharmaceutical Sciences, College of Pharmacy, University of Hawaii at Hilo, Hilo, HI 96720;; 2 University of Hawaii Cancer Center, University of Hawaii at Manoa, Honolulu, HI 96813;; 3 Department of Cell and Molecular Biology, John A. Burns School of Medicine, University of Hawaii at Manoa, Honolulu, HI 96813, USA;; 4 Department of Pediatric Oncology/Hematology, Sophia Children’s Hospital, Erasmus University Medical Center, 3015 GE Rotterdam;; 5 Department of Human Genetics, Academic Medical Center, University of Amsterdam, Amsterdam, The Netherlands;; 6 Department of Cellular and Molecular Physiology, Pennsylvania State University College of Medicine, Hershey, PA 17033, USA

**Keywords:** DFMO, Kaplan-Meier survival plot, metastasis, MYCN, neuroblastoma, ornithine decarboxylase, polyamines, p27^Kip^

## Abstract

Neuroblastoma (NB) is the most common extracranial pediatric tumor. NB patients over 18 months of age at the time of diagnosis are often in the later stages of the disease, present with widespread dissemination, and often possess MYCN tumor gene amplification. MYCN is a transcription factor that regulates the expression of a number of genes including ornithine decarboxylase (ODC), a rate-limiting enzyme in the biosynthesis of polyamines. Inhibiting ODC in NB cells produces many deleterious effects including G_1_ cell cycle arrest, inhibition of cell proliferation, and decreased tumor growth, making ODC a promising target for drug interference. DFMO treatment leads to the accumulation of the cyclin-dependent kinase inhibitor p27^Kip1^ protein and causes p27^Kip1^/Rb-coupled G_1_ cell cycle arrest in MYCN-amplified NB tumor cells through a process that involves p27^Kip1^ phosphorylation at residues Ser10 and Thr198. While p27^Kip1^ is well known for its role as a cyclin-dependent kinase inhibitor, recent studies have revealed a novel function of p27^Kip1^ as a regulator of cell migration and invasion. In the present study we found that p27^Kip1^ regulates the migration and invasion in NB and that these events are dependent on the state of phosphorylation of p27^Kip1^. DFMO treatments induced MYCN protein downregulation and phosphorylation of Akt/PKB (Ser473) and GSK3-β (Ser9), and polyamine supplementation alleviated the DFMO-induced effects. Importantly, we provide strong evidence that p27^Kip1^ mRNA correlates with clinical features and the survival probability of NB patients.

## Introduction

Neuroblastoma (NB) is the most common extracranial pediatric tumor. NB patients diagnosed over 18 months of age are often in the later stages of the disease with widespread dissemination and often possess MYCN tumor gene amplification, a significant predictor of poor outcome (1–4). MYCN is a transcription factor that regulates the expression of a number of genes involved in cell differentiation, proliferation, migration, and invasion (5–9). Ornithine decarboxylase (ODC) is a rate-limiting enzyme in the biosynthesis of polyamines and its expression is regulated by MYCN. Inhibiting ODC in NB cells produces many deleterious effects including G_1_ cell cycle arrest, inhibition of cell proliferation, and decreased tumor growth, making ODC a promising target for drug interference.

Our previous studies have shown that α-difluoromethylornithine (DFMO; also referred to as eflornithine) treatment induced significant accumulation of the cyclin-dependent kinase inhibitor p27^Kip1^ protein and caused p27^Kip1^/Rb-coupled G_1_ cell cycle arrest in MYCN-amplified NB tumor cells (10). In addition, we found that the anti-proliferative effect of DFMO includes p27^Kip1^ phosphorylation at residues Ser10 (nuclear export) and Thr198 (protein stabilization). Furthermore, DFMO activates an opposing pathway that promotes NB cell survival via Akt/PKB (11). The p27^Kip1^ protein, encoded by the CDKN1B gene, is a member of the Cip/Kip family of CDK-binding cell cycle inhibitors, and is well known for its tumor suppressor function. The CDK inhibitor function of p27^Kip1^ has been well characterized and the CDK/cyclin binding domains in regulating this nuclear process are well documented (12–16). Interestingly, recent studies have revealed an additional, cytoplasmic function of p27^Kip1^ in the regulation of cell migration and invasion, and metastasis (17–24). However, the role of p27^Kip1^ in these processes is controversial because it has also been shown to inhibit migration and invasion in some cell types, and promote these processes in other cell types. The phosphorylation of p27^Kip1^ at Ser10 and Thr198 residues has been implicated in cell migration and invasion by regulating the interaction between p27^Kip1^ and the RhoA small GTPases, thereby modulating RhoA activity. In addition, the C-terminal end of p27^Kip1^ was identified as a ‘scatter’ domain involved in cell migration that binds and regulates activity of the Rac1 small GTPase (20,25–27). Finally, p27^Kip1^ has been shown to regulate cell migration by binding the microtubule stabilizing protein, stathmin (17,28). Therefore, the phosphorylation and localization of p27^Kip1^ could control whether this protein regulates cell proliferation or cell migration (19,20,25,27,29–33). Together, these facts demonstrate how p27^Kip1^ regulates multiple roles in the cell depending on its expression, localization, and phosphorylation status.

The aim of the present study was to investigate the clinical relevance of p27^Kip1^ expression in NB and to examine its role in NB migration and invasion. We demonstrate that high p27^Kip1^ mRNA expression in tumors correlates with increased overall survival of NB patients and is predictive of decreased bone and bone marrow metastasis. Furthermore, we examine the mechanism by which polyamines regulate p27^Kip1^, and subsequently modulate NB migration and invasion. Interestingly, the effects of DFMO were significantly greater in NB cells with MYCN overexpression. The results from the present study suggest that both p27^Kip1^ mRNA and p27^Kip1^ protein expression correlate with the survival probability of NB patients by activating mechanisms that not only inhibit tumor growth, but also decrease NB metastasis. Finally, this study presents new evidence of the role of polyamines in cancer, and specifically the malignant progression of NB.

## Materials and methods

### Cell lines and treatment of cultured cells

The human NB cell line MYCN2 (provided by Jason Shohet, TX, USA) were maintained in DMEM (Biosource, Rockeville, MD, USA) containing 10% (v/v) tetracycline-free, heat-inactivated fetal bovine serum (FBS) (Invitrogen, Carlsbad, CA, USA), and hygromycin (100 *μ*g/ml) (34). Cells in early log-phase were seeded and doxycyline (100 ng/ml) was added 3 h before treatment with 5.0 mM α-difluoromethylornithine (DFMO) and incubated for an additional 72 h. NB cells were cultured at 37°C, in a humidified atmosphere containing 5% CO_2_. DFMO was kindly provided by Dr Patrick Woster (Medical University of South Carolina, Charleston, SC, USA). Putrescine (put), spermidine (spd), and spermine (spm) were from Sigma-Aldrich (St. Louis, MO, USA).

### Flow cytometry

Cells were seeded and treated with 5.0 mM DFMO ± 10 *μ*M spermidine or left untreated. Cells were trypsinized, washed twice in phosphate-buffered saline (PBS), counted, and 1–2×10^5^ cells suspended in 0.1 ml of PBS. Cells were stained with 5 *μ*l propidium iodide for 30 min in the dark at room temperature. Assay buffer (0.4 ml) was added, and 5,000 cells analyzed using a FACScan flow cytometry instrument (Becton-Dickinson, San Jose, CA, USA). The CellQuest program (Becton Dickinson) was used for data analysis.

### Western blot analysis

Cell lysates were prepared in RIPA buffer [20 mM Tris-HCl, pH 7.5, 0.1% (w/v) sodium lauryl sulfate, 0.5% (w/v) sodium deoxycholate, 135 mM NaCl, 1% (v/v) Triton X-100, 10% (v/v) glycerol, 2 mM EDTA], supplemented with Complete protease inhibitor cocktail (Roche Molecular Biochemicals, Indianapolis, IN, USA), and phosphatase inhibitors sodium fluoride (NaF) (20 mM) and sodium vanadate (Na_3_VO_4_) (0.27 mM). Western blot analysis was performed as previously described (10). The total protein concentration was determined using the protein assay dye reagent from Bio-Rad Laboratories (Hercules, CA, USA). Cell lysates in SDS-sample buffer were boiled for 5 min and equal amounts of total protein analyzed by 10% SDS-polyacrylamide gel electrophoresis (SDS-PAGE) and western blotting. The antibodies used in this study are: rabbit polyclonal phospho-GSK3-β (Ser9) (1:1,000), rabbit polyclonal phospho-Akt/PKB (Ser473) (1:1,000), rabbit polyclonal anti-tubulin (1:1000) from Cell Signaling Technology, Inc. (Beverly, MA, USA); rabbit polyclonal anti-MYCN (1:500) and rabbit polyclonal anti-p27^Kip1^ (1:500) from Santa Cruz Biotechnology, Inc. (Santa Cruz, CA, USA). Secondary anti-mouse (1:5,000) and anti-rabbit (1:5,000) antibodies coupled to horseradish peroxidase (HRP) were from Amersham Biosciences (Piscataway, NJ, USA). Proteins were detected using the ECL Plus reagents (Amersham Biosciences) and Kodak BioMax XAR film (Fisher Scientific, Pittsburgh, PA, USA). Membranes were stripped at 50°C for 30 min with ECL stripping buffer (62.5 mM Tris-HCl, pH 6.7, 2% SDS, 100 mM 2-mercaptoethanol) and sequentially probed. Quantification was performed as described previously using a Bio-Rad Fluor-S Multi Imager and Quantity One Quantitation Software, Version 4 (Bio-Rad Laboratories).

### Polyamine pool analysis

Intracellular polyamine (putrescine, spermidine, spermine) pools were measured in human MYCN2 cells treated with DFMO using a previously described method (35). The samples were normalized in 0.2 N sodium hydroxide and the amount of total protein per sample was measured using the Bio-Rad assay. Polyamine measurements are presented as nmol per mg of total cellular protein.

### Cell proliferation assay

The CellTiter 96^®^ AQ_ueous_ One Solution Cell Proliferation Assay is a colorimetric method for determining the number of viable cells in proliferation or cytotoxicity assays (Promega, San Luis Obispo, CA, USA). The CellTiter 96^®^ AQueous One Solution Reagent contains the tetrazolium compound MTS [3-(4,5-dimethylthiazol-2-yl)-5-(3-carboxymethoxyphenyl)-2-(4-sulfophenyl)-2H-tetrazolium, inner salt] and was used to determine the proliferation rate of cells treated with DFMO ± putrescine, spermidine or spermine and compared to untreated control cells. Briefly, cells were seeded at a density of 1,000 cells/well on a transparent, flat-bottom 96-well plate, in a total volume of 100 *μ*l. After cell treatments, 20 *μ*l of CellTiter 96 AQueous One Solution Reagent was added to wells, and incubated for 1–4 h at 37°C. The absorbance was measured at 490 nm using a 96-well microplate reader.

### Knockdown experiments with siRNA

Cells were transfected with siRNA from Santa Cruz Biotechnology, Inc. The p27-specific siRNA (sc-29429) used in this study was as follows: sense: AAGUACGAGUGGCAAGAGGUG and antisense: CACCUCUUGCCACUCGUACUU. The scrambled control siRNA (sc-36869) was from Santa Cruz. Briefly, siRNA (40–80 pmol) and Lipofectamine 2000 reagent (10 *μ*l) were diluted, in separate vials, in serum-free DMEM. After 5-min incubation at room temperature, the siRNA and Lipofectamine 2000 were mixed together and incubated at room temperature for an additional 30 min, then added to the cells. The medium was exchanged with DMEM supplemented with 10% FBS after overnight incubation. The cells were analyzed at 48 h post-transfection.

### Wound healing assay

NB cells were grown to confluence and serum-starved overnight. A uniform scratch was placed through the confluent cell monolayer. Cells were washed 3 times and then allowed to settle for 2 h. The width of the wound was measured and recorded as t=0. The cells were then allowed to migrate back into the wounded area, and the closing of the wound was measured over time (t=8–12 h).

### Boyden chamber assay

Migration and invasion assays were performed as outlined in the manufacturer’s protocol (Trevigen, Gaithersburg, MD, USA). Briefly, transwell plates were either coated with 0.7X basement membrane extract (BME) solution and allowed to incubate for 4 h at 37°C in a CO_2_ incubator, or left uncoated. Serum-starved cells (5×10^4^ cells) were seeded into the top chamber in medium without FBS, while medium with FBS was present in the bottom chamber. The cells were incubated for 24 h. The media and remaining cells were aspirated from the top chamber and washed 2 times with 1X wash buffer. The bottom chamber was aspirated and washed 2 times with 1X wash buffer. Calcein-AM in cell dissociation solution was added to the bottom chamber, the cell migration/invasion device re-assembled, and incubated for 1 h. The top chamber was removed and the fluorescence intensity (calcein-AM labeled cells) was measured at 485 nm excitation (520 nm emission) in a plate reader.

### Gene annotation

NCBI Gene_IDs and NCBI Gene Names for the genes analyzed were respectively: ODC: 4953, ODC1 (ornithine decarboxylase) and CDKN1B: 1027, CDKN1B (cyclin-dependent kinase inhibitor 1B; p27, Kip1).

### Affymetrix DNA micro-array hybridization and analysis

The Affymetrix NB tumor dataset NB88 contains the expression profiles of 88 NB tumors with documented genetic and clinical features as previously reported (34). This set is called ‘NB88’. Total RNA was extracted from frozen NBs containing >95% tumor cells, and Affymetrix HG-U133 Plus 2.0 micro-array analysis (Affymetrix, Santa Clara, CA, USA) was performed as described (36). The NB88 set has been deposited for public access in a MIAME-compliant format through the Gene Expression Omnibus (GEO) database at the NCBI website under no. GSE16476. Normalized expression data from the public GEO data-sets for the Jagannathan-100 (GSE19274) and Oberthuer-251 set (E-TABM-38; EMBL-EBI ArrayExpress) series were downloaded and analyzed as described (36). Annotations and clinical data for these series are available from http://www.ncbi.nlm.nih.gov/geo/query/ or http://www.ebi.ac.uk/arrayexpress/ through their GEO or EMBL-EBI ID’s, respectively. Affymetrix probe-sets were selected using the R2 bio-informatic platform (see below). All gene transcript levels were determined from data image files using GeneChip operating software (MAS5.0 and GCOS1.0, from Affymetrix). Samples were scaled by setting the average intensity of the middle 96% of all probe-set signals to a fixed value of 100 for every sample in the data-set, allowing comparisons between micro-arrays. The probe-sets selected for a gene showed the highest expression in samples containing a present call for that gene. The TranscriptView genomic analysis and visualization tool was used to check if the probe-set selected had an anti-sense position in an exon of the gene (http://r2.amc.nl). The Affymetrix probe-sets selected were 200790_at (ODC) and 209112_at (CDKN1B). All analyses were performed using R2, an Affymetrix analysis and visualization platform developed in the Department of Oncogenomics at the Academic Medical Center at the University of Amsterdam. R2 can be accessed at: http://r2.amc.nl.

### Microscopy

For immunofluorescence micrographs, cells were washed twice in PBS, fixed in 4% (w/v) paraformaldehyde for 10 min, and exposed to 0.1% (v/v) Triton X-100 for 3 min. Fixed cells were washed again, blocked in 1% (w/v) bovine serum albumin in PBS for 30 min, and incubated with p27^Kip1^ antibody (1:100) for 1 h. After incubation, cells were washed twice with PBS and incubated for 1 h with anti-rabbit Alexa Fluor 488 antibody (1:5,000). Washed coverslips were mounted using ProLong Gold Antiface mounting medium (Invitrogen) containing 4’,6-diamidino-2-phenylindole and samples were analyzed with a Leica laser-scanning confocal microscope equipped with a digital camera.

## Results

### Expression of p27^Kip1^ in NB tumors

**Figure 1:**
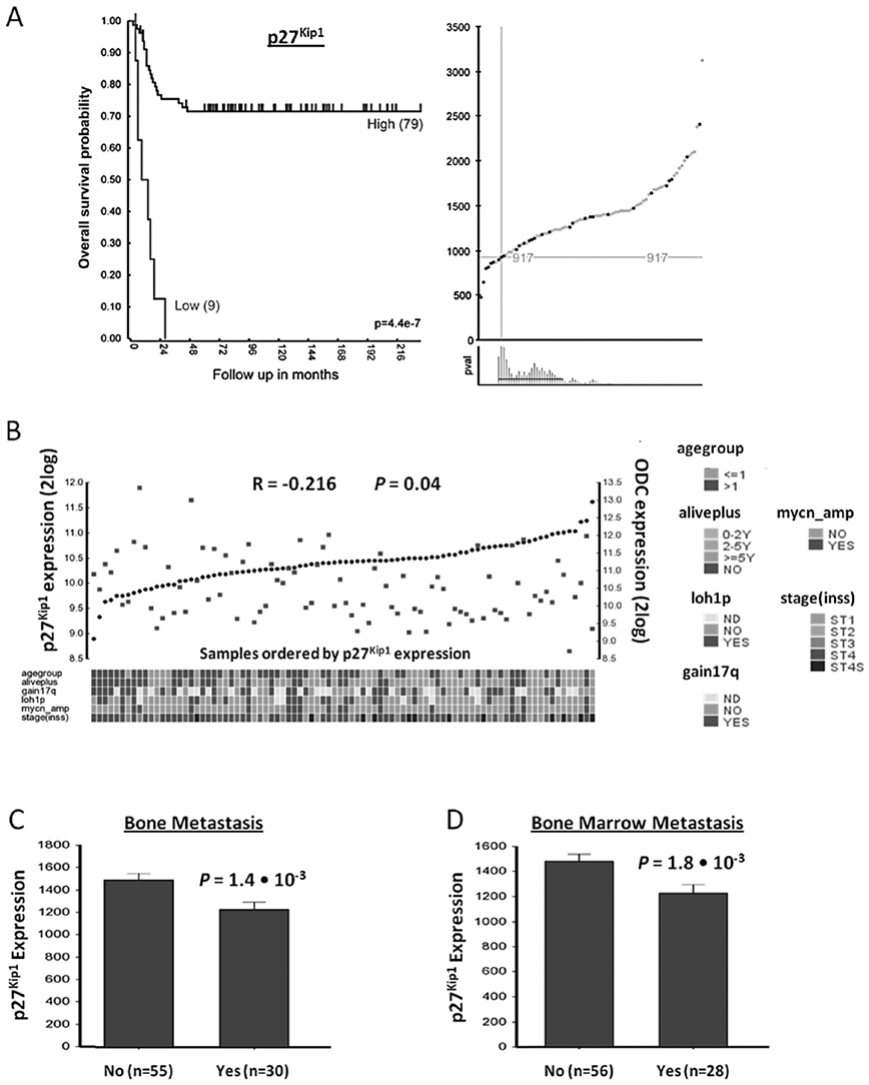
p27^Kip1^ gene expression correlation with patient prognosis, ODC expression, and metastasis in NB. (A) Correlation of p27^Kip1^ gene expression with NB patient survival prognosis. The Kaplan-Meier graph represents the survival prognosis of 88 NB patients based on high or low expression levels of p27^Kip1^ (A, left graph). The survival probability of NB patients (follow-up over 216 months) with low p27^Kip1^ expression is significantly lower than of patients with high p27^Kip1^ expression. For the Kaplan-Meier analysis, the P-values were calculated for all 72 groups tested. For p27^Kip1^, the 9 ‘low’ versus the 79 ‘high’ group represents the most significant P-value (P=4.4×10^−7^), but the P-value was <0.05 for all groups from 8 low/80 high to 30 low/58 high. Also when the 88 tumors were divided using the median or average p27^Kip1^ expression, the P-value was <0.05, showing that p27^Kip1^ expression has a robust correlation with survival as shown in the expression curves and P-value ranges for the Kaplan-Meier graph (A, right graphs). Upper graph, X-axis shows tumors ranked from left to right according to their p27^Kip1^ gene expression level, green dots and red dots represent tumors from patients that were still alive or were dead from disease at the time of this analysis, respectively. Y-axis shows Affymetrix p27^Kip1^ expression levels. Crosshairs mark the expression cut-off used for the grouping shown in the Kaplan-Meier curve. Lower graph, X-axis shows tumor groupings tested. Y-axis shows P-value of each grouping (with a P-value of 1 at the bottom). Red horizontal line represents the P<0.05 cut-off. Statistical analysis was performed with the log-rank test. (B) p27^Kip1^ and ODC expression correlation in NB tumors. Visual representation of p27^Kip1^ and ODC expression in all 88 NB tumors, ranked horizontally from left to right according to their p27^Kip1^ expression. p27^Kip1^ and ODC expression values for each tumor are visualized with black circles and red rectangles, respectively. The correlation between p27^Kip1^ and ODC expression is r = −0.216, with P=0.04 (2log Pearson). Below the graph is the clinical annotation of all 88 tumor samples, the annotation legend is to the right of the graph. (C and D) Correlation of p27^Kip1^ expression levels with NB metastasis. Bone and bone marrow metastasis are significant predictors of poor outcome in NB. (C) Correlation with bone metastasis. Children with NB metastasized to the bone (n=30) show significantly lower p27^Kip1^ tumor expression than infants without metastasis (55 samples; P=1.4×10^−3^, data available for 85 of 88 tumors). (D) Correlation with bone metastasis. Children with NB metastasized to the bone marrow (n=28) show significantly lower p27^Kip1^ tumor expression than infants without metastasis (56 samples; P=1.8×10^−3^, data available for 84 of 88 tumors). Statistical analysis of (C) and (D) was performed using the non-parametric Kruskal-Wallis tests, but for reasons of representation, the bar plots show actual expression values. For expression value calculations in all panels, see Materials and methods.

To examine the expression of p27^Kip1^ (CDKN1B) and its clinical relevance in NB, a set of Affymetrix mRNA expression profiles for 88 NB tumors was prepared (named NB88) using the genome-wide HG-U133 Plus 2.0 platform. The genetic and clinical features were available from patient files for all 88 NB tumors (Academic Medical Center at the University of Amsterdam). Using Kaplan-Meier scan analysis, a correlation between p27^Kip1^ mRNA expression and NB patient survival was found (Fig. 1A). While the most significant P-value was calculated when the NB88 set was divided into a group of 79 with high, and of 9 with low expression of p27^Kip1^ mRNA, the expression of p27^Kip1^ mRNA was also significantly indicative for survival in other groupings (Fig. 1A). Survival of patients with high p27^Kip1^ expression (n=79) was ∼70% for up to 216 months, while that for patients with low p27^Kip1^ expression (n=9) dropped to 0% within 30 months (P=4.4×10^−7^).

Next, we examined whether there was a correlation between p27^Kip1^ and ODC mRNA expression in the NB88 tumor set. Fig. 1B shows a visual representation of p27^Kip1^ and ODC mRNA expression measured with Affymetrix profiling for every tumor in the NB88 set. An inverse relation between p27^Kip1^ and ODC expression (r = −0.216, P=0.04) was observed. This suggests that ODC expression and intracellular polyamine levels may inhibit p27^Kip1^ expression or decrease p27 ^Kip1^ mRNA stability.

NB metastasis to bone and bone marrow is predictive of poor patient outcome. We therefore investigated whether p27^Kip1^ expression correlates with bone and bone marrow metastasis in the NB88 set. Indeed, there was a significantly higher expression of p27^Kip1^ in tumors without bone metastasis (P=1.4–1.8×10^−3^ Kruskal-Wallis t test) or bone marrow metastasis (P=2.2×10^−3^ Kruskal-Wallis t test) (Fig. 1C and D). These results suggest that p27^Kip1^ expression correlates with decreased metastasis and increased survival of NB patients.

### DFMO-induced polyamine depletion inhibits NB proliferation and induces G_1_ cell cycle arrest

**Figure 2:**
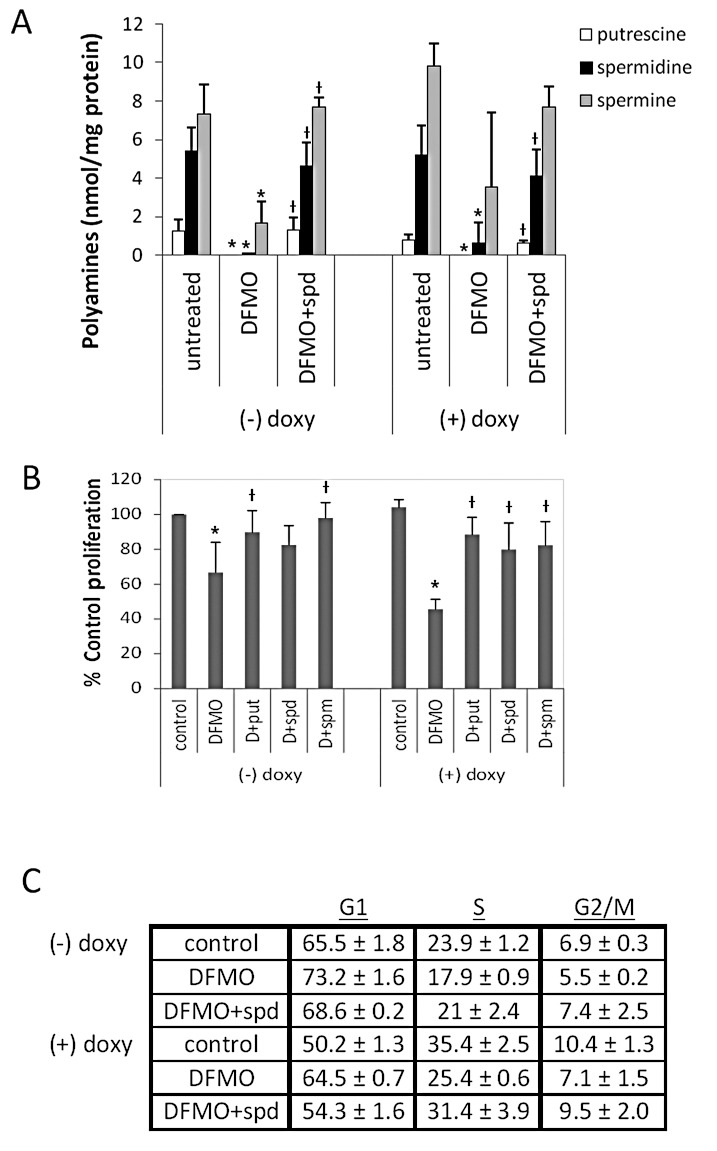
DFMO inhibits polyamine biosynthesis, cell proliferation, and induces G_1_ cell cycle arrest in tetracycline-inducible MYCN overexpressing NB cells (MYCN2). (A) Cells were treated with doxycycline ± 5 mM DFMO ± 10 *μ*M spermidine or left untreated for 72 h, and intracellular polyamine levels were measured. DFMO treatment depleted intracellular polyamine levels, and supplemental spermidine in culture media reversed the effects of DFMO. (B) Cells were treated with doxycycline ± 5 mM DFMO ± 10 *μ*M putrescine, 10 *μ*M spermidine or 10 *μ*M spermine, or left untreated for 72 h, and cell proliferation was measured using the MTS assay. DFMO significantly inhibited proliferation in NB cells with and without MYCN overexpression. Supplementing external media with polyamines reversed the effects of DFMO. Results of (A) and (B) are represented as mean ± SD, n=3. ^*^Statistically significant difference between values obtained from DFMO-treated vs. untreated cells. ^†^Statistically significant difference between values obtained from DFMO-treated cells and cells treated with both DFMO and spermidine (P<0.05). (C) Cells were treated with doxycycline ± 5 mM DFMO ± 10 *μ*M spermidine or left untreated for 72 h, and flow cytometry was performed with propidium iodide to quantify the percentage of cells in each phase of the cell cycle (G_1_, S, and G_2_/M). DFMO increased the percentage of cells in the G_1_ phase of the cell cycle, and supplementing external media with spermidine reversed the effects of DFMO. Results are represented as mean ± SD, n=3. Doxy, doxycyline; put, putrescine; spd, spermidine; spm, spermine.

Our previous studies have shown that DFMO depletes intracellular polyamine pools in MYCN gene-amplified LAN-1 and NMB-7 NB cells (10). To examine the effect of DFMO in NB cells with and without MYCN overexpression, we used MYCN2 NB cells which contain a doxycycline-inducible MYCN transgene. The cells were grown either in the presence or absence of doxycycline [with or without MYCN overexpression, called MYCN2 (+) or MYCN2 (−), respectively], and then treated with 5 mM DFMO ± 10 *μ*M spermidine, or left untreated for 72 h. The intracellular polyamine levels were measured to determine the efficacy of 5 mM DFMO on MYCN2 cells. DFMO depleted intracellular putrescine (put), spermidine (spd) and spermine (spm) levels significantly in both MYCN2 (+) and MYCN2 (−) cells (Fig. 2A). Supplementing the medium with exogenous spd during DFMO treatment restored intracellular polyamines in both cell lines to levels comparable to that of control cells treated with vehicle (water) without DFMO (Fig. 2A), confirming the effect of DFMO on polyamine biosynthesis. The results clearly show that 5 mM DFMO effectively decreased intracellular polyamine levels in MYCN2 cells, independent of MYCN expression.

To determine whether MYCN overexpression influences the efficacy of DFMO in NB, cell proliferation was measured in MYCN2 cells utilizing the tetrazolium based MTS assay. While DFMO treatment significantly inhibited the proliferation in both MYCN2 (−) and MYCN2 (+) compared to untreated cells (Fig. 2B), the inhibitory effect of DFMO was significantly enhanced when MYCN was overexpressed in NB cells. Polyamine (put, spd or spm) supplementation attenuated this DFMO effect in both cell lines (Fig. 2B).

To further examine the influence of DFMO on NB cell proliferation in the absence or presence of MYCN, we determined the cell cycle distribution of MYCN2 (−) and MYCN2 (+) cells. The results revealed that DFMO caused a perturbation in the cell cycle distribution of these cells by increasing the fraction of MYCN2 (−) and MYCN2 (+) cells in the G_1_ phase of the cell cycle and by decreasing the fraction of MYCN2 (−) and MYCN2 (+) cells in the S and G_2_/M phases of the cell cycle, compared to control untreated cells (Fig. 2C). The inhibitory effects of DFMO were greater in MYCN2 (+) cells compared to MYCN2 (−). The addition of spd reversed the effects of DFMO, indicating that these effects were due to polyamine depletion. These results show that DFMO induces G_1_ cell cycle arrest, an effect that is more pronounced in NB cells with MYCN over-expression.

### DFMO inhibits cell migration in MYCN2 cells

**Figure 3:**
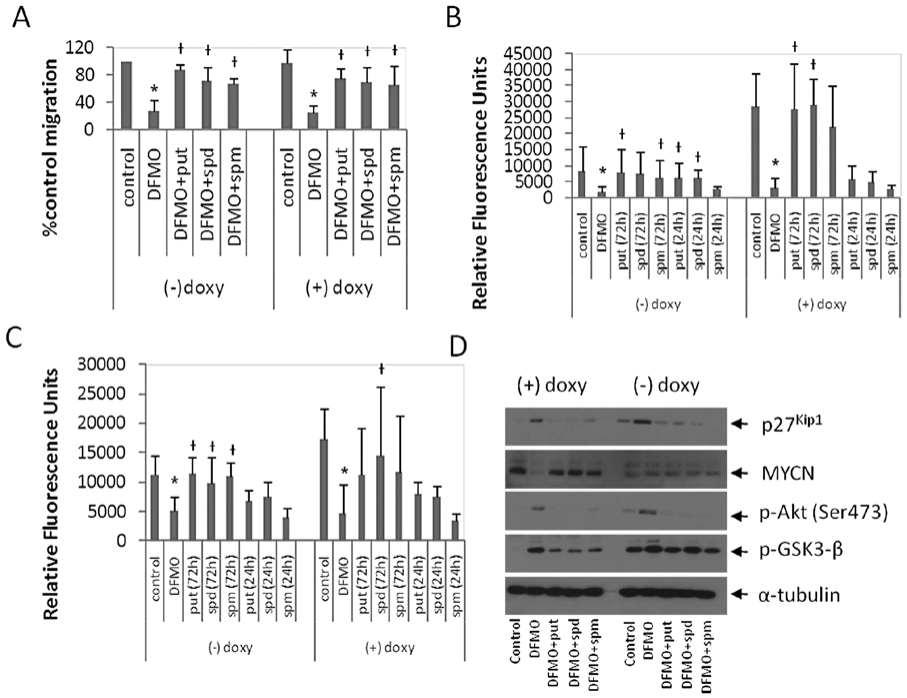
DFMO inhibits cell migration and invasion, increases p27^Kip1^ expression, and increases the phosphorylation of Akt (Ser473) and GSK3-β (Ser9). (A) MYCN2 cells were treated with or without doxycycline and 5 mM DFMO ± 10 *μ*M putrescine, 10 *μ*M spermidine or 10 *μ*M spermine for 72 h and the wound healing assay was performed. DFMO inhibited cell migration. Supplementing the external media with putrescine, spermidine or spermine reversed the effects of DFMO. Results are represented as mean ± SD, n=3. (B and C) MYCN2 cells were treated with doxycycline ± 5 mM DFMO. The external medium was supplemented with ± 10 *μ*M putrescine, 10 *μ*M spermidine or 10 *μ*M spermine or left untreated for 72 h of DFMO treatment or the last 24 h of DFMO treatment. Transwell migration (B) and invasion (C) assays were performed. DFMO inhibited NB migration and invasion. Supplementing external media with polyamines for 72 h of DFMO treatment reversed the effects of DFMO. Supplementing external media with polyamines for the last 24 h of DFMO treatment only partially reversed the effects of DFMO. (A–C) Results are represented as mean ± SD, n=3 in duplicates. ^*^Statistically significant difference between values obtained from DFMO-treated vs. untreated cells. ^†^Statistically significant difference between values obtained from DFMO-treated cells and cells treated with both DFMO and putrescine, spermidine or spermine (P<0.05). (D) MYCN2 cells were treated with or without doxycycline and 5 mM DFMO ± 10 *μ*M putrescine, 10 *μ*M spermidine or 10 *μ*M spermine for 72 h. DFMO treatment induced p27^Kip1^ accumulation, downregulation of MYCN expression, and increased the phosphorylation of Akt (Ser473) and GSK3-β (Ser9). Tubulin was used as a loading control. Analysis was performed in three independent experiments (n=3). Doxy, doxycyline; put, putrescine; spd, spermidine; spm, spermine.

To examine the regulation of NB cell migration by polyamines and MYCN, MYCN2 (−) and MYCN2 (+) cells were treated with DFMO ± put, spd or spm, or left untreated for 72 h, and NB migration was measured using the wound healing assay (Fig. 3A). As early as 6–8 h, a significant difference between cell migration of untreated control cells compared to DFMO-treated cells was observed. DFMO inhibited cell migration by 73 and 72% in MYCN2 (−) and MYCN2 (+) cells, respectively (Fig. 3A). Supplementing the medium with polyamines (put, spd or spm) alleviated the effects of DFMO in MYCN2 (−) cells and MYCN2 (+) cells (Fig. 3A). These results suggest that DFMO-induced polyamine depletion exerted an inhibitory effect on cell migration in MYCN2 cells, and supplementing DFMO treatment with any of the three polyamines was able to partially reverse this inhibitory effect.

To extend these results, we examined the effects of DFMO on NB invasion using the Boyden chamber transwell assay. MYCN2 cells were treated with DFMO or left untreated for 72 h. The culture medium of the DFMO-treated cells was supplemented with polyamines (put, spd or spm), either for 72 h of DFMO treatment or for only the last 24 h of the DFMO treatment. In MYCN2 (−) cells, DFMO inhibited migration by 56% compared to untreated control cells (Fig. 3B). The presence of exogenously added polyamines for 72 h attenuated this effect to levels comparable to untreated control cells. The addition of polyamines for the last 24 h of the 72-h DFMO incubation period only partially reversed the DFMO effects. Interestingly, NB migration was ∼1.5-fold higher in MYCN2 (+) cells than in MYCN2 (−) cells. In MYCN2 (+) cells DFMO inhibited migration by 73% compared to untreated cells (Fig. 3B). The addition of polyamines to the medium of DFMO-treated cells during the 72 h of treatment attenuated the DFMO effect to levels comparable to untreated cells (Fig. 3B). Supplementing DFMO treatment with polyamines for the last 24 h of DFMO treatment only partially reversed the DFMO effects on NB migration (Fig. 3B).

To determine whether polyamines also regulate NB invasive migration, the transwell assay was repeated. However, in this experiment, the porous filter which separates the top and bottom chambers of the assay plate was coated with extracellular matrix molecules. In order to examine the role of MYCN in this process, MYCN2 (−) and MYCN2 (+) cells were treated as described above. In MYCN2 (−) cells, DFMO inhibited invasion by 77% compared to untreated cells (Fig. 3C). Polyamine (put, spd, spm) supplementation for 72 h of the DFMO treatment reversed the drug-induced effect (Fig. 3C). Supplementing DFMO-treatment with polyamines for the last 24 h of treatment partially attenuated the DFMO effects (Fig. 3C). MYCN overexpression increased NB invasion by ∼3.5-fold compared to NB cells without MYCN overexpression. In MYCN2 (+) cells, DFMO inhibited NB invasion by 89% compared to untreated cells (Fig. 3C) and polyamine supplementation for 72 h of DFMO-treatment reversed the drug-induced effect (Fig. 3C). Supplementing DFMO-treated cells with polyamines for the last 24 h of DFMO treatment did not reverse the drug-induced effects (Fig. 3C). These results suggest that polyamines regulate NB invasion and that DFMO suppresses invasiveness most effectively in MYCN overexpressing NB cells.

To identify the cellular signaling pathways involved in DFMO action, MYCN2 (+) and MYCN2 (−) cells were treated with DFMO ± polyamines (put, spd or spm), or left untreated for 72 h. Whole cell lysates were prepared and analyzed by Western blotting. Doxycycline-induced MYCN overexpression was confirmed by the increased intensity of the 67-kDa band representing the MYCN protein (Fig. 3D). Remarkably, DFMO treatment significantly decreased MYCN protein levels in MYCN2 (+) cells but not in MYCN2 (−) cells that express only low levels of MYCN. DFMO also induced the accumulation of p27^Kip1^ protein (Fig. 3D). The DFMO-induced downregulation of MYCN and accumulation of p27^Kip1^ were reversed by the addition of polyamines (Fig. 3D).

As p27^Kip1^ is a downstream target of Akt/PKB (37,38), the effect of DFMO on Akt/PKB was also examined. DFMO treatment indeed induced an increase in Akt/PKB phosphorylation at Ser473, indicative of Akt/PKB activation (Fig. 3D). DFMO also induced an increase in glycogen synthase kinase-3β (GSK3-β) phosphorylation at Ser9, a downstream target of Akt/PKB (Fig. 3) (39). Polyamine supplementation reversed the effects of DFMO on the phosphorylation of these signaling proteins (Fig. 3D). Together, these results suggest that p27^Kip1^ is involved in the regulation of polyamine-dependent NB migration and/or invasion and that the Akt/PKB-GSK3-β pathway is also involved in these regulatory processes.

### DFMO-induced inhibition of NB migration involves p27^Kip1^

**Figure 4:**
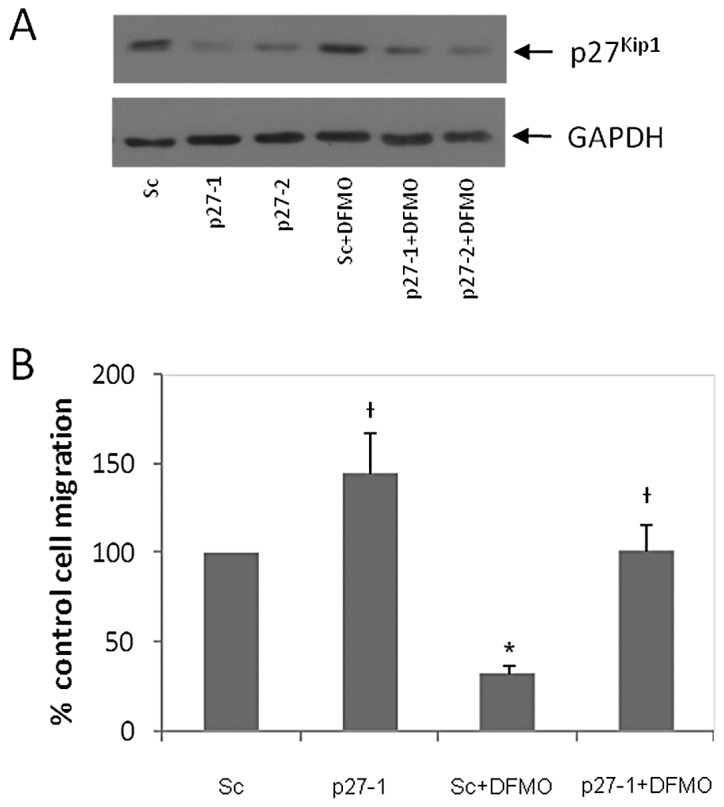
Downregulation of p27^Kip1^ increased NB migration. NB cells were transfected with siRNA specific for p27^Kip1^ or scrambled (Sc) sequence. p27-1 represents transfection of cells with 40 pmol siRNA and p27-2 represents 80 pmol siRNA. Control cells were transfected with 80 pmol scrambled siRNA. (A) Whole cell lysates were analyzed by western blotting for p27^Kip1^ expression. GAPDH was used as a loading control. (B) Wound healing assays. Untreated and DFMO-treated cells transfected with p27^Kip1^ siRNA had a higher migration rate than cells transfected with scrambled siRNA. ^*^Statistically significant difference between values obtained from DFMO-treated vs. untreated cells, transfected with scrambled siRNA. ^†^Statistically significant difference between values obtained from DFMO-treated cells transfected with scrambled siRNA and DFMO-treated and untreated cells transfected with p27^Kip1^ siRNA (P<0.05). Data are represented as mean ± SD (n=2).

DFMO-induced polyamine depletion was previously shown to induce p27^Kip1^ protein accumulation, and p27^Kip1^ phosphorylation at Ser10 and Thr198 (10,11). To determine the role of p27^Kip1^ in DFMO-induced inhibition of NB migration, p27^Kip1^ specific siRNA was used to downregulate p27^Kip1^ protein expression in NB cells. Scrambled siRNA was used as a control. The p27^Kip1^ siRNA (p27-1 and p27-2 represent 40 and 80 pmol siRNA, respectively) and scrambled siRNA (designated ‘sc’) were transfected into MYCN2 (−) cells. These transfected cells were either exposed to DFMO or left untreated for 72 h. Western blot analysis of whole cell lysates revealed that p27^Kip1^ siRNA downregulated p27^Kip1^ protein levels compared to sc siRNA (Fig. 4A). As expected, DFMO increased p27^Kip1^ levels in cells transfected with sc, but not in those transfected with p27-1 or p27-2 siRNA (Fig. 4A). Cells transfected with p27^Kip1^ or sc control siRNA were also used to measure cell migration using the wound healing assay. Fig. 4B shows that downregulation of p27^Kip1^ increased cell migration by 45% compared to the scrambled control. DFMO treatment inhibited cell migration by 68% in scrambled control cells. However, p27^Kip1^ downregulation completely reversed DFMO-induced inhibition of cell migration to that of untreated (no DFMO) scrambled cells (Fig. 4B). These results show that p27^Kip1^ plays a key role in regulating NB migration.

### Expression and subcellular localization of p27^Kip1^ in NB cells treated with DFMO

**Figure 5:**
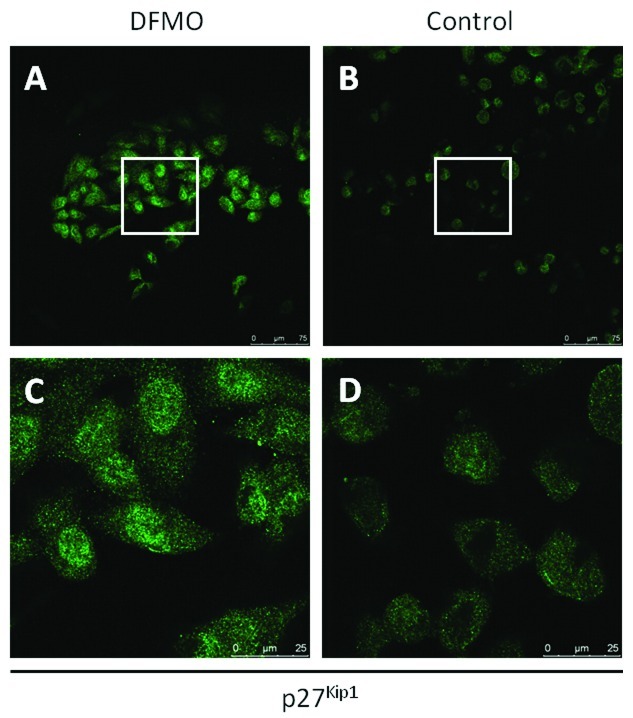
DFMO treatment induces p27^Kip1^ accumulation in the nucleus and cytoplasm. DFMO-treated cells (A) and untreated cells (B) were fixed and immunostained fluorescently for p27^Kip^ and analyzed by confocal microscopy. The white squares in the lower power images (A and B) are magnified below (C and D). Data are representative of three independent experiments (n=3).

To further examine the role of p27^Kip1^ in NB cell migration and invasion, the subcellular localization of p27^Kip1^ was examined in NB cells. MYCN2 (+) cells were treated with DFMO or left untreated for 72 h. Confocal laser microscope analysis of DFMO-treated and untreated NB cells revealed that DFMO treatment resulted in an increase of p27^Kip1^ protein levels in NB cells compared to untreated cells. Importantly, DFMO treatment led to the accumulation of p27^Kip1^ protein in both the nucleus and cytoplasm (Fig. 5), suggesting that cytoplasmic p27^Kip1^ may function to inhibit NB migration and invasion, a function that may be separate and distinct from its role in cell cycle regulation.

## Discussion

**Figure 6:**
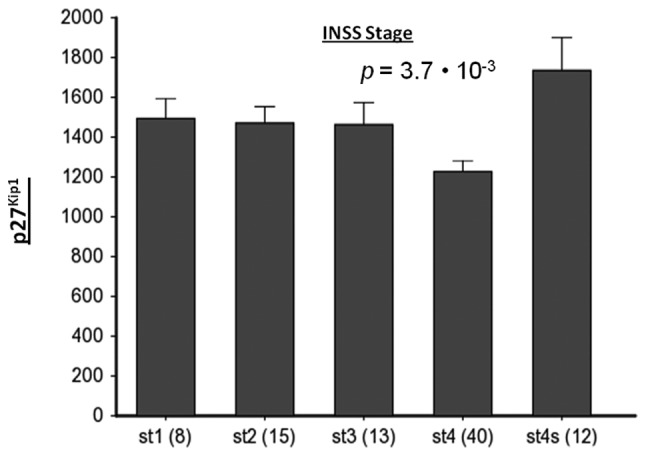
Stage 4 NB has significantly lower p27^Kip1^ mRNA expression than other NB stages. The results shown are for the NB88 set described in this study, the P-value is for a Kruskal-Wallis t-test. In addition, significantly lower p27^Kip1^ expression in stage 4 NB than in other NB stages was found in the Jagannathan-100 (P=7.1×10^−3^) and Oberthuer-251 (P=9.3×10^−5^) series in the public domain. For data set details and analyses see Materials and methods.

This study analyzed the role of polyamines and p27^Kip1^ in NB metastasis. First, we determined the clinical relevance of p27^Kip1^ in NB. High p27^Kip1^ expression correlated with increased patient survival in the NB88 set presented in this study. This result was confirmed in the only other publicly available NB tumor cohort with Kaplan-Meier survival data, the Oberthuer-251 set (results not shown), and these findings are consistent with those from an earlier study (40). We also investigated the correlation between p27^Kip1^ expression and metastasis of the bone and bone marrow. The expression of p27^Kip1^ correlated with fewer occurrences of bone and bone marrow metastasis. Interestingly, NB patients in stages 1, 2 and 3 have higher p27^Kip1^ expression than those in stage 4, which is the metastasizing stage (Fig. 6). Finally, we observed an inverse correlation between p27^Kip1^ and ODC mRNA expression suggesting that ODC and possibly intra-cellular polyamines regulates metastasis through a process that involves modulation of p27^Kip1^ expression.

Our previous studies have shown that the ODC inhibitor DFMO depletes intracellular polyamine levels, induces p27^Kip1^ protein accumulation and G_1_ cell cycle arrest, downregulates MYCN protein, but also promotes cell survival by modulating the phosphorylation of Akt/PKB via the PI3K/Akt signaling pathway in MYCN-amplified NB cells (10,11). In the present study, we found that DFMO inhibits the migration of NB cells. The results from the wound healing assay suggested that MYCN overexpression had no effect on NB migration and DFMO inhibited migration to the same degree, regardless of the MYCN status. The results from the transwell migration assay suggested that MYCN overexpression enhanced NB migration, and the inhibitory effect of DFMO was augmented in MYCN2 (+) cells. In addition, MYCN overexpression increased NB invasion by 3.5-fold and DFMO was much more potent at inhibiting invasion in MYCN2 (+) cells than MYCN2 (−) cells. The different effects observed in the wound healing assay compared to the transwell assay may be due to the differences in the assay design. The wound healing assay in two-dimensional cell culture allows for chemokinetic migration, or random migration, but not chemotaxis. In contrast, the design of the transwell assay with chemoattractant in the bottom chamber allows for both chemo-kinetic and chemotactic migration. The results from this set of experiments showed that polyamines promote NB migration and invasion, and DFMO is a potent inhibitor of these processes. In addition, MYCN overexpression significantly increased NB invasion and DFMO inhibited migration and invasion more effectively in MYCN over-expressing cells relative to controls.

Interestingly, each individual polyamine appeared to be equally effective at regulating NB proliferation, migration, and invasion. In addition, the reversal of the DFMO-induced effects by polyamines appeared to be a time-dependent process. However, other polyamine-metabolizing enzymes are still active and the addition of any polyamines can be converted or retro-converted into the other polyamines. Therefore, it is still unclear whether one particular polyamine plays a larger role than the other polyamines in regulating NB proliferation, migration or invasion.

Next, we examined the role of p27^Kip1^ in NB migration and invasion and discovered that downregulation of p27^Kip1^ expression attenuated the inhibitory effect of DFMO on NB migration and invasion. Furthermore, DFMO induced translocation of p27^Kip1^ from the nucleus to the cytoplasm of NB cells. The results from these experiments suggest that polyamines regulate NB migration and invasion by modulating the expression and localization of p27^Kip1^, and that DFMO inhibits NB migration and invasion by inducing p27^Kip1^ accumulation. Our previous results showed that DFMO induces Ser10 phosphorylation of p27^Kip1^, which has been shown to be a nuclear export signal (11). Phosphorylation at this site was also shown to induce nuclear accumulation in some cell types (41). The results from this study showed that DFMO induces p27^Kip1^ accumulation in both the nucleus and cytoplasm suggesting a dual role for p27^Kip1^. While nuclear p27^Kip1^ is a well-established cell cycle regulator, nuclear and/or cytoplasmic p27^Kip1^ may play a role in regulating cell migration and invasion.

Previous studies have shown that polyamines regulate cell migration and metastasis in other cell types (42–50). The present study examined the role of polyamines in NB migration and invasion and provides evidence that p27^Kip1^ plays a key role in regulating these processes, in addition to its well-characterized role as a cell cycle regulator. In other studies, DFMO has been shown to regulate these processes via p21^Cip1^. However, in NB cells we have shown that DFMO effects are mediated via p27^Kip1^. Therefore, p27^Kip1^ expression is not only a prognostic indicator of NB, but also an indicator for NB metastasis. This is particularly interesting since although p27^Kip1^ is a well-known tumor suppressor gene, its mRNA expression in a number of different cancer types is not lower in cancerous than in corresponding normal tissues (data not shown). In NB, however, p27^Kip1^ expression is in fact lower in patients with stage 4, meta-static NB than those patients with lower stage, non-metastatic disease. This was not just found for the NB set described in this study, but also for two other NB tumor sets in the public domain (Fig. 6). In addition, this study revealed that there is an inverse relationship between p27^Kip1^ and ODC expression suggesting that the function of p27^Kip1^ in tumor progression, metastasis and overall survival may be regulated by intracellular polyamines. We also showed that DFMO exhibits anti-migratory properties, and it will be interesting to see whether this is also true of NB metastasis, *in vivo*. Importantly, the potency of DFMO on NB migration and invasion was greater in NB cells with MYCN overexpression.

NB cells stabilize MYCN protein, for example, by inhibition of MYCN degradation. Akt/PKB-mediated inactivation of GSK3-β (through phosphorylation on its Ser9 residue) prevents GSK3-β from phosphorylating MYCN on Thr58, which normally leads to proteasomal degradation of MYCN (51). Our results show that DFMO can reduce MYCN protein levels in MYCN2 (+) cells, in spite of Akt/PKB activation, as shown by p27^Kip1^ accumulation, providing a molecular rationale for the use of DFMO in NB tumors with high MYCN expression. Together, these results suggest that DFMO may be particularly effective as part of a combination treatment to prevent metastasis of NB, and for the treatment of advanced stage, NB patients with MYCN-amplification. Recent *in vivo* studies with NB tumor-bearing mice using the transgenic TH-MYCN model revealed significant antitumor effects of DFMO (52,53). Importantly, a phase I human clinical trial with DFMO alone and combined with etoposide in relapsed/refractory NB is near completion (ClinicalTrials. gov Identifier NCT01059071) and a phase II preventative trial with DFMO in patients with high-risk NB in remission is now open for participant enrollment at several Neuroblastoma and Medulloblastoma Research Consortium (NMTRC) Children’s Hospitals throughout the US (ClinicalTrials.gov Identifier NCT01586260).
